# Dually Modified Cellulose as a Non-Viral Vector for the Delivery and Uptake of HDAC3 siRNA

**DOI:** 10.3390/pharmaceutics15122659

**Published:** 2023-11-23

**Authors:** Juliana Hülsmann, Henry Lindemann, Jamila Wegener, Marie Kühne, Maren Godmann, Andreas Koschella, Sina M. Coldewey, Thomas Heinze, Thorsten Heinzel

**Affiliations:** 1 Institute of Biochemistry and Biophysics, Center for Molecular Biomedicine, Friedrich Schiller University Jena, Hans-Knöll-Straße 2, 07745 Jena, Germany; juliana.huelsmann@uni-jena.de (J.H.); marie.kuehne@med.uni-jena.de (M.K.); maren.godmann@uni-jena.de (M.G.); 2 Institute for Organic Chemistry and Macromolecular Chemistry, Center of Excellence for Polysaccharide Research, Friedrich Schiller University Jena, Humboldtstraße 10, 07743 Jena, Germany; henry.lindemann@uni-jena.de (H.L.); andreas.koschella@uni-jena.de (A.K.); thomas.heinze@uni-jena.de (T.H.); 3 Department of Anesthesiology and Intensive Care Medicine, Jena University Hospital, Am Klinikum 1, 07747 Jena, Germany; jamila.wegener@med.uni-jena.de (J.W.); sina.coldewey@med.uni-jena.de (S.M.C.); 4 Septomics Research Center, Jena University Hospital, Albert-Einstein-Straße 10, 07745 Jena, Germany; 5 Center for Sepsis Control and Care, Jena University Hospital, Am Klinikum 1, 07747 Jena, Germany; 6 Jena Center for Soft Matter (JCSM), Friedrich Schiller University Jena, Philosophenweg 7, 07743 Jena, Germany

**Keywords:** guanylated cellulose, biotin, polyplexes, non-viral vectors, siRNA delivery, HDAC3

## Abstract

RNA interference can be applied to different target genes for treating a variety of diseases, but an appropriate delivery system is necessary to ensure the transport of intact siRNAs to the site of action. In this study, cellulose was dually modified to create a non-viral vector for *HDAC3* short interfering RNA (siRNA) transfer into cells. A guanidinium group introduced positive charges into the cellulose to allow complexation of negatively charged genetic material. Furthermore, a biotin group fixed by a polyethylene glycol (PEG) spacer was attached to the polymer to allow, if required, the binding of targeting ligands. The resulting polyplexes with *HDAC3* siRNA had a size below 200 nm and a positive zeta potential of up to 15 mV. For N/P ratio 2 and higher, the polymer could efficiently complex siRNA. Nanoparticles, based on this dually modified derivative, revealed a low cytotoxicity. Only minor effects on the endothelial barrier integrity and a transfection efficiency in HEK293 cells higher than Lipofectamine 2000^TM^ were found. The uptake and release of the polyplexes were confirmed by immunofluorescence imaging. This study indicates that the modified biopolymer is an auspicious biocompatible non-viral vector with biotin as a promising moiety.

## 1. Introduction

Since the approval of ONPATTRO^®^ in 2018, which was the first FDA-approved siRNA, the potential of therapeutic siRNA became apparent [1]. The advantage of the siRNA mechanism relies on its potential application to any desired target and the possibility to act on targets where approved small molecules are missing [2]. RNA-based therapeutics require an adequate delivery system because of their poor stability due to their 2′-hydroxy group, which can be easily cleaved by enzymes, resulting in no therapeutic effect [3]. In this study, histone deacetylase 3 (HDAC3) siRNA was delivered using a dually modified cellulose as a non-viral vector. Recent studies have shown that an imbalance between histone acetyltransferases (HATs) and HDACs leads to an increase of deacetylated histones and, therefore, to an inability of the transcription machinery to transcribe genes, resulting in impaired cellular processes, signal transduction, and human diseases [4,5]. These findings were confirmed through an altered gene expression, induced by hypoacetylation in diseases like cancer and sepsis [4]. Thus, restoration of the acetylation levels and the original state through histone deacetylase inhibitors (HDACis) is a promising approach for treating a variety of diseases. Well-known HDACi like valproic acid (VPA) and vorinostat (SAHA) often exhibit low target specificity, a short serum half-life, and short dose intervals. siRNA can overcome these obstacles due to its higher target specificity, fewer side effects, and the opportunity to act on non-druggable targets, like HDAC3 [6]. HDAC3 is known to be a part of the inflammatory pathways in macrophages, as HDAC3 knockout macrophages are unable to activate inflammatory genes [7]. In the kidney, HDAC3 is essential for kidney development, and it is expressed in nephron precursors and glomerular podocytes [8]. Upregulation of HDAC3 is found in injured podocytes, renal fibrosis, chronic kidney diseases (CKDs), and various cancers, indicating that HDAC3 is a promising target in several diseases [9].

To deliver siRNA successfully into cells, a carrier with certain properties is essential: (i) a high complexation ability for siRNA, (ii) protection against enzymatic degradation, and (iii) facilitating the cellular uptake as well as the intracellular release of the cargo [10]. In the past, viral vectors were often used, but, in many cases, they display high immunogenicity and toxicity [11,12]. Synthetic non-viral vectors are often based on polyethylenimine and polymethacrylamides, but also natural polymers like dextran or cellulose can be used [13,14]. Biopolymers, especially polysaccharides, are known for their non-toxicity, biocompatibility, and biodegradability [15]. Cellulose and its derivatives are widely used in the pharmaceutical field as coating and filling material in tablets and are approved by the European Medicine Agency (EMA) as well as by the US Food and Drug Administration (FDA) [16]. The synthesis of a cationic group-bearing cellulose allows its usage as a non-viral vector.

The non-viral vector mimics the properties of cell-penetrating peptides or the HIV-TAT protein through their arginine-rich or amino group-containing side chains [17]. The cationic groups allow the complexation of genetic material like plasmid DNA (pDNA), siRNA, and messenger RNA (mRNA) [18]. In general, guanidine is a strong organic base; hence, it is permanently positively charged, independent of the pH value of the environment, and, therefore, it occurs as guanidinium cation. It has been suggested that the guanidinium group initiates strong nucleic acid binding and cellular uptake by its highly basic nature (pK_a_ ≈ 13.4) and a specific bidentate hydrogen-bonding formation with anionic phosphate or sulphate groups located at the surface of cell membranes due to high cationic, delocalized charge densities [19,20,21,22]. On the other hand, the guanidinium group is held responsible for cyto- and hemotoxicity due to its strong cationic nature, whereas the combination of guanidinium structures and biopolymer backbones is an opportunity to increase its biocompatibility, reduce toxic effects, and use its advantageous properties for non-viral vectors [23]. Eswaran et al. have already shown such an approach, where they generated biocompatible, non-toxic nanohydrogels based on PEO-PPO micelles, cross-linked with NIPAM and acrylonitrile. The resulting nanohydrogels contained polyamines, which were able to complex and deliver DNA in vitro and in vivo [24,25]. On the one hand, they showed a remarkable biocompatibility, and, on the other hand, the binding capacity of the polyamines was reduced compared to the binding capacity of the guanidinium groups, emphasizing the specificity of the guanidinium group. In 1991, 1*H*-pyrazole-1-carboxamidine hydrochloride (PCA) was developed as a guanylation reagent for amino groups. PCA is able to react with primary and secondary amines under mild reaction conditions in various organic solvents and water without side reactions to form guanidinium structures [26,27]. For example, amino acids have been modified for peptide chemistry. In the field of polysaccharides, amino celluloses are suitable substrates. They are produced via the conversion of cellulose *p*-toluenesulfonic acid esters with various molecules containing amino groups. In case of ethylenediamine, the substituent can also be positively charged. The nucleophilic replacement reaction is selective for the primary hydroxy group [28]. The remaining hydroxy groups can then be used for further side-chain modifications that further influence the properties of the vector, e.g., polyethylene glycol can reduce toxicity, a hydrophobic moiety can favor cellular uptake, and fluorination leads to a higher serum stability [13,29,30].

Biotinylation is an interesting research approach for an effective tissue-specific drug delivery. The strong physical interaction of biotin with streptavidin can be utilized to couple cell-specific antibodies to nanoparticles and polyplexes [31,32]. In previous research, the carboxylic acid group of biotin was used for its direct attachment to the polymer or to combine it with a linker/spacer. Biotinylated, antibody fragment-binding polyplexes showed cellular uptake and high knockdown efficiency of luciferase activity in vitro and significant anti-tumor effects on PSCA-positive tumors in vivo compared to non-modified polyplexes [33].

In this study, 6-deoxy-6-(2-aminoethyl) amino cellulose (EDAC) was modified with a guanidinium group and a PEG-biotin moiety to offer the possibility to couple targeting molecules like antibodies and peptides. This dually modified cellulose derivative served as a carrier for *HDAC3* siRNA. The resulting polyplexes, made only of dually modified cellulose and *HDAC3* siRNA, were first characterized without any targeting moiety and compared to Lipofectamine 2000^TM^. The physicochemical properties of the polyplexes were evaluated with regard to particle size, zeta potential, and binding affinity as well as stability under endolysosomal conditions. The uptake of the nanoparticles was studied via fluorescence microscopy. Biological evaluation focused on cell viability, endothelial barrier integrity, gene knockdown efficiency, and protein levels of HDAC3 in kidney cells, regarding its described clinical relevance [9].

## 2. Materials and Methods

### 2.1. Synthesis of 6-Deoxy-6-(2-aminoethyl) Amino Cellulose

The 6-deoxy-6-(2-aminoethyl) amino cellulose was prepared according to previously published literature [28].

DS_EDA_: 0.54 (EDAC1); 0.81 (EDAC2, EDAC3).
^1^H NMR (250 MHz, D_2_O, δ): 7.93 (H_arom_), 7.61 (H_arom_), 5.29–3.09 (H1-6), 2.64 (H7,8).
^13^C NMR (63 MHz, D_2_O, δ): 129.67 (C_arom_), 125.69 (C_arom_), 102.67 (C1), 78.70–73.43 (C2, C3, C4, C5), 60.34 (C6), 49.12 (C6_NH_), 47.25 (C7), 39.30 (C8).
EA: C 40.73; H 7.02; N 8.02; S 0.59; DS_EDA_ = 0.54.

### 2.2. Synthesis of 6-Deoxy-6-(2-guanidiniumethyl) Amino Cellulose Chloride (Typical Example, GEDAC2)

PCA (3.97 g, 27.1 mmol, Sigma Aldrich, Darmstadt, Germany) and a 1.0 M NaOH solution (10.83 mL) were added to EDAC1 (DS = 0.54, 1.0 g, 5.41 mmol) which was dissolved in water (100 mL). The reaction mixture was allowed to react at 80 °C for 24 h under stirring. The polymer was precipitated in EtOH (1000 mL), collected by filtration, and washed 3 times in EtOH (200 mL). The polymer was dissolved in water (50 mL) and freeze-dried. The polymer was washed 3 times with diethyl ether (200 mL) and dried in a vacuum at 40 °C for 24 h.

Yield: 0.64 g.
^1^H NMR (250 MHz, D_2_O, δ): 5.62–2.77 (H1-6; H7,8).
^13^C NMR (63 MHz, D_2_O, δ): 161.6 (C10), 157.4 (C9), 102.7 (C1), 83.8–65.7 (C2, C3, C4, C5), 70.9 (C6_Guan._), 60.2 (C6_OH_), 48.6 (C6_NH_), 46.7 (C7), 39.7 (C8), 37.9 (C8′).
EA: C 35.42%; H 6.48%; N 14.39%; Cl 9.95%.

### 2.3. Biotinylation of 6-Deoxy-6-(2-aminoethyl) Amino Cellulose

GEDAC4 (0.3 g, 1.1 mmol) was dissolved in 10 mL H_2_O, followed by the addition of biotin-PEG4-NHS-ester (0.056 g, 0.096 mmol, MedChemExpress, Monmouth Junction, NJ, USA) and Na_2_CO_3_ (0.1 g, 1.2 mmol), and the mixture was allowed to react at RT for 24 h under stirring. The polymer was purified by precipitation in 2-propanol (200 mL) and collected by filtration. The polymer was washed 3 times in 2-propanol (200 mL). The product was redissolved in water (50 mL) and freeze-dried.

Yield: 0.25 g.
^1^H NMR (500 MHz, D_2_O, δ): 8.16 (NH_Biotin_), 7.83 (NH_Biotin_), 5.33–3.23 (H1-6, H7), 2.73 (H8), 2.53 (H23), 1.91 (H26), 1.68 (H25), 1.52 (H24).
^13^C NMR (101 MHz, D_2_O, δ): 177.1 (C11, C22), 161.6 (C10), 157.4 (C9), 102.7 (C1), 81.2 (C4) 75.2–73.4 (C2, C3, C5), 69.9 (C15–20), 69.2 (C14), 68.3 (C13), 62.4 (C29), 60.6 (C6, C30), 55.6 (C27), 49.0 (C6_NH_), 47.2 (C7), 46.8 (C7′), 41.6 (C28), 39.7 (C8), 38.1 (C8′), 35.8 (C23), 28.2 (C12), 28.0 (C24, C26), 25.4 (C25).
EA: C 36.96%, H 6.50%, N 15.22%, S 0.61%.

NMR spectra were acquired on a Bruker Avance I 250 and Avance III 400 spectrometer with 16 scans for ^1^H NMR and up to 21,000 scans for ^13^C NMR spectra. D_2_O was taken as NMR solvent for the NMR spectra (DEUTERO GmbH, Kastellaun, Germany). The elemental analysis was performed on a Euro Vector EA 3000 CHNS Analyzer (HEKAtech, Wegberg, Germany).

The DS of EDA were calculated from the elemental analysis according to Equation (1):

(1) $${\mathrm{DS}}_{\mathrm{EDA}}=\frac{\frac{\%N}{100\%}{\;\times \;M}_{\mathrm{RU}}\;}{M_{N}{\;\times \;n}_{N\;\mathrm{sub}\;}-\frac{\%E}{100\%}{\;\times \;(M}_{\mathrm{EDA}}-{\;M}_{H2O})}$$

with

%N = percentage of nitrogen;
M_RU_ = molar mass of RU (162.14 g mol^−1^);
M_N_ = molar mass of nitrogen (14.00 g mol^−1^);
n_N sub_ = number of N in substituent;
M_EDA_ = molar mass of EDA (60.10 g mol^−1^);
M_H2O_ = molar mass of removed OH from repeating unit plus H from EDA (18.01 g mol^−1^).

(2) $${\mathrm{DS}}_{\mathrm{Biotin}}=\frac{\frac{\%S}{100\%}{\;\times \;(M}_{\mathrm{RU}}{+(\mathrm{DS}}_{\mathrm{EDA}}{\;\times \;M}_{\mathrm{EDA}}-{\;\mathrm{DS}}_{\mathrm{EDA}}\;{-\;M}_{\mathrm{OH}})+(\mathrm{DG}\;\times {\;M}_{G}-\;\mathrm{DG}\;-{\;M}_{H}))}{M_{S}-\frac{\%S}{100\%}{\;\times \;(M}_{\mathrm{Biotin}-\mathrm{PEG}4}-{\;M}_{H})}$$

with

%S = percentage of sulfur;
M_RU_ = molar mass of RU (162.14 g mol^−1^);
M_EDA_ = molar mass of EDA (60.10 g mol^−1^);
M_H_ = molar mass of hydrogen (1.001 g mol^−1^);
M_S_ = molar mass of sulfur (32.06 g mol^−1^);
M_G_ = molar mass of the substituted part of guanidinium chloride (79.01 g mol^−1^);
M_Biotin-PEG4_ = molar mass of Biotin-PEG4 (474.60 g mol^−1^);

### 2.4. Conductometric Titration

A defined mass of the GEDAC samples was dissolved in water and was acidified to a pH value of 2.8 with a 0.1 M HCl solution. The mixture was titrated with 50 mL of a 0.01 M NaOH solution with a speed of 0.75 mL min^−1^ under stirring using the titrator TitroLine^®^ 7800 (SI Analytics^®^, Mainz, Germany). All samples were measured 3 times to determine the arithmetic means.

The DG value was determined according to Equation (3):

DG = DS_EDA_ − DS_EDAn_ (3)

with

DS_EDA_ = DS of EDA;
DS_EDAn_ = new DS of EDA after guanylation.
DS_EDAn_ was calculated according to Equation (4):

(4) $${\mathrm{DS}}_{\mathrm{EDAn}}=\frac{{\mathrm{DS}}_{\mathrm{EDA}}{\;\times \;c\;\times \;M}_{G}\;\times \;V\;-{\;\mathrm{DS}}_{\mathrm{EDA}}{\;\times \;c\;\times \;M}_{H}\;\times \;V\;-{\;\mathrm{DS}}_{\mathrm{EDA}}{\;\times \;c\;\times \;M}_{\mathrm{EDA}}\;\times \;V\;-{\;\mathrm{DS}}_{\mathrm{EDA}}{\;\times \;c\;\times \;M}_{\mathrm{OH}}{\;\times \;V+\mathrm{DS}}_{\mathrm{EDA}}{\;\times \;c\;\times \;M}_{\mathrm{EDA}}{\;\times \;V+c\;\times \;M}_{\mathrm{RU}}\;\times \;V}{{c\;\times \;M}_{G}\;\times \;V\;-\;c\;\times {\;M}_{H}\;\times \;V\;-{\;c\;\times \;M}_{\mathrm{EDA}}{\;\times \;V+2\;\times \;m}_{P}}$$

with

c = concentration of NaOH (0.01 M);
M_G_ = molar mass of the substituted part of guanidinium chloride (79.01 g mol^−1^);
V = calculated titration volume of aqueous NaOH for non-guanylated amino moieties;
M_H_ = molar mass of hydrogen (1.001 g mol^−1^);
M_EDA_ = molar mass of EDA (60.10 g mol^−1^);
M_OH_ = molar mass of OH (17.00 g mol^−1^);
M_RU_ = molar mass of RU (162.14 g mol^−1^);
m_p_ = sample mass.

### 2.5. Size-Exclusion Chromatography (SEC) Measurement 

The SEC (Jasco Deutschland GmbH, Pfungstadt, Germany) measurement of the GEDAC samples was processed in a mixture with H_2_O, 0.1% TFA, and 0.1% NaCl. The flow rate was 1 mL min^−1^, and the polymer was detected with a refractive index detector (RI-930). Pullulan served as the standard for molar mass calibration.

### 2.6. Polyplex Formation

The polyplexes were prepared using the dually modified cellulose and Silencer^®^ Validated histone deacetylase 3 (HDAC3) siRNA (sense: 5′GCAACCCAGCUGAACAACAtt 3′ and antisense: 5′ UGUUGUUCAGCUGGGUUGCtc 3′) (Ambion^®^, Cambridge, UK). The nitrogen to phosphate ratio (N/P ratio) was calculated as the ratio of the nitrogen (N) containing cellulose to the *HDAC3* siRNA, which contains negatively charged phosphate (P) groups. Both chemicals were solved in nuclease-free water (ThermoFisher^TM^, Darmstadt, Germany). The siRNA solution had a concentration of 200 ng mL^−1^ and the polymer stock solution of 5 mg mL^−1^. For the polyplex formation of the calculated N/P ratios, the required volumes of both solutions were diluted in a saline buffer (150 mM NaCl and 10 mM HEPES; pH 7.4; both Carl Roth, Karlsruhe, Germany) or other media of choice. After 10 min of incubation at RT, equal volumes of the solutions were mixed to a desired volume, whereby the polymer solution was added to the siRNA solution. Subsequently, the solution was vortexed for 10 s and incubated again for 10 min before use. Polyplexes were freshly prepared prior to each experiment.

### 2.7. Binding Capacity

To investigate the dually modified cellulose’s binding capacity, a Quant-iT™ microRNA Assay (ThermoFisher^TM^) was performed, according to the manufacturer’s instructions. To compare the influence of the dilution media on the binding capacity, four different media were chosen. Nuclease-free water (ThermoFisher^TM^), saline buffer (see polyplex formation), Opti MEM^TM^, and RPMI 1640 + 10% fetal calf serum (FCS) (all Gibco, ThermoFisher^TM^) were compared. 

Polyplex solutions were pipetted in a black 96-well plate (Fluotrac 200, Greiner Bio-One, Frickenhausen, Germany) and incubated together with 200 µL of the Quant-iT-reagent for 10 min at 450 rpm and RT. Afterwards, the fluorescence signal was captured using the Tecan^®^ Spark 20M (485 nm excitation; 530 nm emission; Tecan Group AG, Maennedorf, Switzerland). The dilution medium served as the blank control, while the uncomplexed siRNA diluted in the dilution medium was set as 100%. The binding capacity of the different N/P ratios was calculated as a percentage of the free siRNA after subtracting the blank value. Polyplexes of branched poly(ethylene imine) (bPEI, 10 kg mol^−1^; Polysciences Europe GmbH, Eppelheim, Germany) and *HDAC3* siRNA at N/P ratio 20 were utilized as a positive control. Data are shown as percentages (mean ± SEM), *n* = 2, with 4 technical replicates.

### 2.8. Particle Size and Zeta Potential Measurements

Particle size measurements were carried out using dynamic light scattering (DLS) with the Malvern Zetasizer Nano ZS (Malvern Instruments GmbH, Herrenberg, Germany), and a nanoparticle tracking analysis (NTA) was performed with the NanoSight^®^ LM10 (Malvern Instruments GmbH). The polyplexes were tested using N/P ratios between 2 and 40. Both measurements were performed at 25 °C. For the DLS measurements, the polyplexes were prepared in 100 µL saline buffer. For the NTA measurements, the same samples were diluted with bidistilled water at a ratio of 1:3 to a total volume of 300 µL. A red laser light at 638 nm and a tracking time of 30 s and 5 repetitions per sample were chosen as the experimental settings for the NTA.

The Zetasizer Nano ZS (Malvern Instruments GmbH) was used to measure the zeta potential in bidistilled water. The undiluted sample was measured at RT using a high-concentration capillary cell (ZEN1010, Malvern Instruments GmbH). The results of the Zetasizer Nano ZS were evaluated as triplicates using the Malvern software 6.20. All experiments were performed thrice (mean ± SEM).

### 2.9. Endolysosomal Stability of Polyplexes

The endolysosomal stability of polyplexes was investigated after the formation of polyplexes in nuclease-free water (ThermoFisher^TM^), according to Haladjova et al. [34]. The pH value of the polyplex solution was adjusted to pH 4 with hydrochloric acid (Carl Roth) to simulate the acidic environment in the endolysosome. The polyplex solutions were incubated for 24 h, 48 h, and 72 h at 37 °C to mimic transfection conditions. After incubation, the particle size was determined via NTA, as previously described. The experiment was independently repeated once. Data are shown as mean ± SEM.

### 2.10. In Vitro Cell Culture

HEK293 cells (CRL-1537, ATCC-American Type Culture Collection, Manassas, VA, USA) or HEK293T cells (DSMZ-German Collection of Microorganisms and Cell Cultures GmbH, Braunschweig, Germany) and L929 mouse fibroblasts (ACC 2, DSMZ) were grown in Roswell Park Memorial Institute (RPMI) 1640 medium containing 10% fetal calf serum (FCS) and 1% Penicillin/Streptomycin (P/S) (all Gibco, ThermoFisher^TM^). HeLa cells (DSMZ) were cultured in RPMI 1640 medium, supplemented with 10% FCS and 1% P/S. Human microvascular endothelial cells (HMEC-1; ATCC, Manassas, VA, USA) were cultured as described previously [35]. Endothelial barrier measurement was assessed with passages less than 10. All cell lines were cultured at 37 °C, 5% CO_2_, and 95% relative humidity.

### 2.11. Cell Viability Assays

To test the polymer’s cell viability, a CellTiter-Glo^®^ Luminescent Cell Viability Assay (Promega Corporation, Madison, WI, USA) was performed, according to the manufacturer’s protocol. The assay was performed using the L929 mouse fibroblast cell line. Cells were seeded in a white 96-well plate (Greiner Bio-One) at 8500 cells per well. After 24 h of incubation, a dilution series from 3.9 µg mL^−1^ to 500 µg mL^−1^ of the polymer was prepared in RPMI 1640 (+10% FCS), and 100 µL of each concentration was added to the wells. Subsequently, the cells were incubated for further 24 h. After one day of incubation, 100 µL of the CellTiter-Glo^®^ reagent was added, following 2 min of shaking at 450 rpm and 10 min of incubation without shaking. Luminescence was determined with the Tecan Spark 20M (Tecan Group AG). A thiomersal 0.02% solution (Caelo, Hilden, Germany) served as a positive control, and untreated cells were set as 100% control. RPMI 1640 supplemented with 10% FCS served as the blank value and was subtracted from all the measured values. The final cell viability was calculated by setting the measured values in correlation to the untreated control. Cell viability values below 70% were defined as toxic, according to DIN EN ISO 10993-5 [36]. This assay was repeated twice with eight technical replicates, and data are shown as percentages (mean ± SEM).

### 2.12. Barrier Integrity Measurement Based on Electric Cell-Substrate Impedance Sensing (ECIS^TM^)

The impact of free *HDAC3* siRNA (0.5 ng µL^−1^), polymer (same polymer amount as N/P ratio 10), and polyplexes bearing *HDAC3* siRNA of N/P ratios 2, 5, 10, 20, and 40 on the endothelial barrier of HMEC-1 was evaluated using the cell-substrate impedance sensing (ECIS^TM^) Zθ system (Applied Biophysics Inc., Troy, NY, USA). Measuring in multiple frequency mode, barrier integrity and cell viability was detected by a resistance measurement at 4 kHz and capacitance measurement at 64 kHz. For this, 96W10idf ECIS Cultureware^TM^ Arrays (Applied Biophysics Inc., Troy, NY, USA) were cleansed with 12 mg mL^−1^ L-Cysteine (Sigma Aldrich, St. Louis, MO, USA) and precoated with 1 mg mL^−1^ human fibronectin (Sigma Aldrich, St. Louis, MO, USA) and 1.25 mg mL^−1^ murine collagen IV (Corning, Corning, NY, USA). Stabilization and cell-free measurement was performed at least 1 h prior to cell seeding. HMECs were seeded at a density of 100,000 cells mL^−1^. Half of the medium was replaced every 24 h, and cells were cultivated for 75 h until full confluence of the well and a steady-state resistance for at least 12 h were achieved. After 75 h, cells were stimulated with polyplexes with N/P ratios of 2, 5, 10, 20, and 40 and a final *HDAC3* siRNA concentration of 0.5 µg mL^−1^ in technical and biological triplicates. The measurement continued for 72 h. For analysis, time was resampled to 600 s and the medium control was subtracted from the resistance values. Statistics were performed using GraphPad Prism 7.05 by Kruskal–Wallis and Dunn’s multiple comparison test to the untreated cell control. *p*-values below 0.05 were considered significant.

### 2.13. siRNA Transfection and Real-Time Quantitative PCR

For the gene expression analysis, 250,000 HEK293 cells per well were seeded in a 12-well plate (Greiner Bio-One) and preincubated for 24 h. The polyplexes were prepared in Opti-MEM^TM^. For transfection, 1000 ng of *HDAC3* siRNA was used per sample. The polyplexes were prepared as described above. Lipofectamine 2000^TM^ (ThermoFisher^TM^) served as a positive control. Silencer™ GFP (*eGFP*) siRNA (Ambion^®^) was used as a negative control together with Lipofectamine 2000^TM^. Cells without any additives were used for the normalization of the gene expression. After preincubation, the cells were washed twice with phosphate-buffered saline. Fresh FCS-containing RPMI 1640 medium was added with a total volume of 100 µL of polyplexes per well and incubated for 72 h. After this time, the medium was aspirated, cells were harvested, and RNA isolation was performed using the TRIzol™ reagent (Invitrogen^TM^), according to the manufacturer’s protocol. cDNA synthesis was carried out with the RevertAid First Strand cDNA synthesis kit with oligo(dT)18 primers (ThermoFisher^TM^). A total of 2 µg RNA was used for cDNA synthesis.

*HDAC3* mRNA levels were determined via real-time quantitative PCR (RT-qPCR) on the QuantStudio^TM^ 3 Real-Time PCR system (ThermoFisher^TM^). The PowerUp™ SYBR™ Green Master Mix (ThermoFisher^TM^) was used for signal detection, and the ribosomal protein S2 (*Rps2*) was utilized as housekeeping gene. The following primers were utilized (Biomers, Ulm, Germany):

*HDAC3* forward, 5′-GAGTTCTGCTCGCGTTACACAG-3′-;
*HDAC3* reverse, 5′-CGTTGACATAGCAGAAGCCAGAG-3′;
*Rps2* forward, 5′-GCACCAGGTTCAAGGCATTTG-3′;
*Rps2* reverse, 5′-TCTTGTTCCCCCAGTAGCCT-3′.

mRNA levels were calculated using the 2^−∆∆Ct^ method. The experiment was repeated twice in triplicates, and the results are shown as a mean ± SEM (n = 3).

### 2.14. Western Blot

For the Western blot analysis, HEK293T cells were seeded in a 6-well plate with a quantity of 625,000 cells per well. The controls and the transfection protocol were kept the same as for the gene expression analysis. Cells were harvested 72 h after transfection. The resulting cell pellets were resuspended in NP-40 buffer (150 mM NaCl, NP40 1%, SDS 0.1%, 50 mM Tris-HCl (pH 8.0)) supplemented with 0.2% protease inhibitor cocktail, 50 mM NaF, 100 μM NaVO_3_, 10 mM nicotinamide (NAM), and 0.01 μM trichostatin A (TSA). Afterwards, the suspension was incubated for 30 min on ice and subsequently sonicated using the Digital Sonifier 450 (Branson Ultrasonics Corporation, Danbury, CT, USA). The sonicated samples were centrifuged at 14,000 rpm for 10 min at 4 °C. To determine the protein concentration, the BCA Protein Assay Kit (ThermoFisher^TM^) was used, following the manufacturer’s instructions. For SDS-polyacrylamide gel electrophoresis, 15 µg protein per sample was loaded on a 12% acrylamide gel. Electrophoretic separation took place in a vertical electrophoresis chamber (Bio-Rad Laboratories Inc., Hercules, CA, USA) for 80 min at 20 mA. For the Western blot analysis, the proteins were transferred to a polyvinylidene fluoride (PVDF) membrane (Carl Roth) using transfer buffer (250 mM glycine, 25 mM Tris, 0.1% SDS, 20% Ethanol, all Carl Roth) in the Mini Trans-Blot^®^ Cell Chamber (Bio-Rad, Feldkirchen, Germany) for 90 min at 150 mA. The membrane was blocked for at least 1 h in 5% dry milk in phosphate-buffered saline (PBS). Mouse anti-vinculin (Bio-Rad, #MCA465GA, 1:10,000) and rabbit anti-HDAC3 (Abcam^®^, Cambridge, UK, #ab16047, 1:1000) were used as primary antibodies. Incubation lasted overnight at 4 °C. The incubation with secondary antibodies took place for 1 h at RT with the following antibodies: HRP-conjugated goat anti-rabbit IgG (Santa Cruz Biotechnology, Dallas, TX, USA, #sc2004; 1:2000) or HRP-conjugated goat anti-mouse IgG (Santa Cruz Biotechnology, #sc2005; 1:5000). For signal detection, the Westar Nova 2.0 Kit (Cyanagen, Bologna, Italy) was used, according to the manufacturer’s protocol, using the FUSION solo S system (Vilber Lourmat, Eberhardzell, Germany). The signal evaluation was performed with the Fusion Capt Advance Solo 7 S 17.04a software (Vilber Lourmat). Therefore, the bands of HDAC3 were normalized to the vinculin bands, and the untreated control served as the 100% value. The experiment was repeated twice. Data are shown as a mean ± SEM (*n* = 3).

### 2.15. Immunofluorescence (IF)

For immunofluorescence, 100,000 HeLa cells were seeded in RPMI 1640 (+10% FCS and +1% P/S) on gelatine-coated coverslips (12 mm diameter) and incubated for 24 h. Polyplexes were prepared as previously described, and a siRNA Silencer™ FAM-labelled Negative Control No. 1 siRNA (ThermoFisher^TM^) was used. Cells were washed with PBS, and fresh medium was added together with the polyplexes and incubated for 90 min. Between each of the following incubations, the cells were washed twice with PBS. After the incubation with the polyplexes, the cells were fixated for 30 min with 4% paraformaldehyde (Carl Roth) in PBS. Subsequently, the cells were permeabilized for 5 min with 0.05% Triton X-100 (Carl Roth) in water at RT. For actin staining, a methanol-based rhodamine-phalloidin (ThermoFisher^TM^) solution was used and incubated for 45 min in the dark. For nuclei staining, 300 nM DAPI (4′,6-diamidino-2-phenylindole, Carl Roth) was used and incubated for 8 min in the dark, following washing with PBS twice. The coverslips were transferred to slides using the Fluoromount-G™ Mounting Medium (ThermoFisher^TM^).

Pictures were taken using the Nikon eclipse Ti microscope (Nikon Europe B.V., Amstelveen, The Netherlands). The pictures represent the average of 3 independent experiments.

### 2.16. Statistical Analyses

The software GraphPad Prism 8.0.2. (GraphPad Software, San Diego, CA, USA) and Microsoft Excel (Microsoft Cooperation, Redmond, DC, USA) were used for statistical analyses. Outliers were removed using Grubb’s test (α = 0.05). An unpaired *t*-test was applied, unless stated otherwise, and data are shown as mean ± SEM. The *p*-value was set at *p* < 0.05 (* *p* ≤ 0.05, ** *p* ≤ 0.01, *** *p* ≤ 0.001, **** *p* ≤ 0.0001).

## 3. Results and Discussion

### 3.1. Guanylation of 6-Deoxy-6-(2-aminoethyl) Amino Cellulose

**Figure 1:**
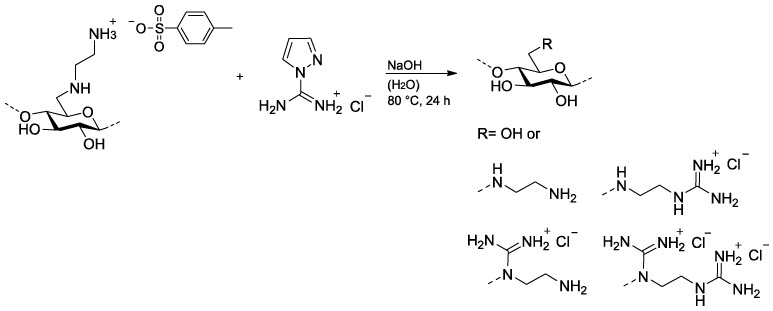
Guanylation of 6-deoxy-6-(2-aminoethyl) amino cellulose with 1*H*-pyrazole-1-carboxamidine hydrochloride in aqueous NaOH at 60 or 80 °C for 24 h.

Previously published EDACs with a DS_EDA_ of 0.54 (EDAC1) and 0.81 (EDAC2 and EDAC3) were allowed to react with 5 mol of PCA per mol modified repeating unit (RU) in aqueous NaOH at 60 or 80 °C for 24 h (Figure 1) [28]. Sodium hydroxide is required to deprotonate the ammonium groups to the reactive amino groups. 6-Deoxy-6-(2-guanidiniumethyl) amino cellulose chlorides (GEDACs), with degree-of-guanylation (DG) values of 0.25 (GEDAC1), 0.33 (GEDAC2), 0.62 (GEDAC3), and 0.69 (GEDAC4) as determined by conductometric titration (Table 1, Figure S1), were synthesized. An increase in the reaction temperature results in increased DG values from 0.25 to 0.33. The conversion of EDAC2 with a DS_EDA_ of 0.81 at 80 °C resulted in a DG of 0.62. The reproducibility of the guanylation was shown by the preparation of the sample GEDAC4 having a DG of 0.69.

### 3.2. NMR Spectroscopic Characterization of 6-Deoxy-6-(2-guanidiniumethyl) Amino Cellulose Chloride

**Figure 2:**
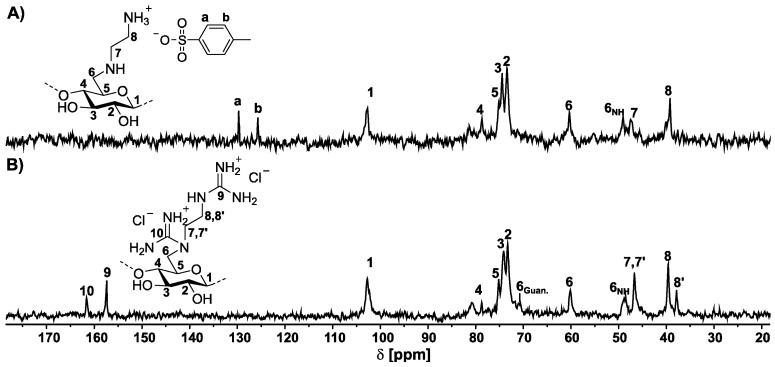
^13^C NMR spectrum of (**A**) 6-deoxy-6-(2-aminoethyl) amino cellulose (EDAC1, DS_EDA_ = 0.54) and (**B**) 6-deoxy-6-(2-guanidiniumethyl) amino cellulose chloride (GEDAC2, DS_EDA_ = 0.54; DG = 0.33) in D_2_O.

The GEDAC samples were characterized by 1D- and 2D-NMR spectroscopic methods. The ^13^C NMR spectrum of GEDAC1 reveals a signal of the guanidinium carbon at 157.4 (C9) ppm and carbon signals of the ethylene moiety at 47.1 (C7′) and 41.5 (C8′) ppm (Figure S2). The signals corresponding to the modified repeating unit of the cellulose derivative appear at 102.5 (C1), 80.0–60.3 (C2, C3, C4, C5, and C6), and 48.6 (C6_NH_) ppm. A signal assigned as the EDAC1 substituent at 39.6 ppm was caused by the incomplete guanylation of the amino groups. The sample GEDAC2 exhibits a further guanidinium carbon signal at 161.1 (C10) ppm in the ^13^C NMR spectrum that results from the guanylation of the secondary amine due to an increase of the reaction temperature from 60 to 80 °C. The guanylation of the secondary amine influenced the chemical shift of C8′ and C6_NH_ and slightly of C7′, leading to signals of C6_Guan._, C7′′, and C8′′. The carbon signal of C6_Guan._ is shifted to 70.9 and C8′′ to 37.9 ppm. The signal of C7′′ is slightly shifted and is overlapped by signals from the EDA substituent and the mono-guanylated substituent. The characteristic signals for the C-atoms of the substituent at 47.5 (C7) and 39.3 (C8) ppm and the substituted position C6_NH_ at 49.1 ppm are visible in the ^13^C NMR spectrum. In comparison, the signals of the RU are not shifted and appear at 102.7 (C1), 81.3–73.4 (C2, C3, C4, and C5), and 60.4 (C6) ppm (Figure 2). The carbon signals were assigned by HSQC DEPT and HMBC NMR spectroscopy (Figures S3 and S4). The chemical shifts are independent of the DG.

### 3.3. Biotinylation of 6-Deoxy-6-(2-guanidiniumethyl) Amino Cellulose

**Figure 3:**
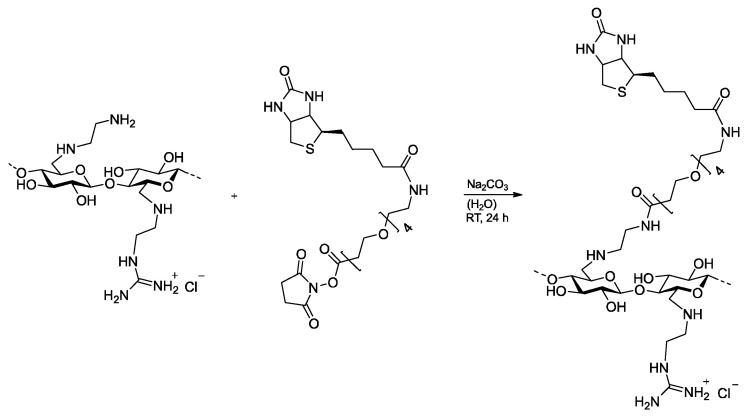
Biotinylation of 6-deoxy-6-(2-guanidiniumethyl) amino cellulose with polyethylene glycole-4-biotin-NHS ester in water for 24 h at RT.

Due to the incomplete guanylation of the EDAC samples, they contain residual amino groups for functionalization with biotin as an anchor group for the targeting units. The sample GEDAC4 was chosen for the biotinylation due to the highest DG. Therefore, the reactive polyethylene glycol-4 (PEG4)-biotin-NHS ester was utilized to attach the molecule by amid linkage at the terminal amino groups to the polymer. The conversion was carried out in water in the presence of Na_2_CO_3_ for 24 h at RT (Figure 3). PEG4 should serve as a spacer to enable better accessibility of the streptavidin protein.

**Figure 4:**
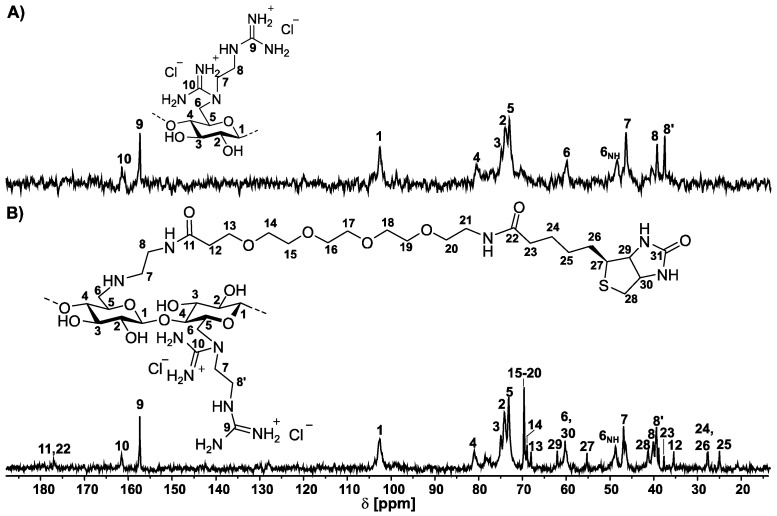
^13^C NMR spectra of (**A**) 6-deoxy-6-(2-guanidiniumethyl) amino cellulose (GEDAC4, DS_EDA_ = 0.81, DG = 0.69) and (**B**) GEDAC4-PEG4-biotin adduct (DS_EDA_ = 0.76, DG = 0.69, DS_EDA-biotin_ = 0.05) in D_2_O.

The reaction was carried out at a molar ratio of 0.09 mol PEG4-biotin-NHS ester per 1.0 mol modified RU, so that, statistically, every polymer chain was modified with a biotin molecule. For the calculation, the mass average molar mass (M_w_) was determined by size-exclusion chromatography. The M_w_ of 79,728 g mol^−1^ was used for the calculation of the degree of polymerization (DP) of 302. The content of the biotin-functionalized EDA moieties was calculated from the sulfur content. The calculation resulted in a functionalization of a DS_EDA_ with PEG4-biotin of 0.05. The polymer has a M_w_ of 73,253 g mol^−1^, which means that the polymer has a DP of 255 and, in the mean average, the polymer carries 12 biotin molecules. The smaller M_w_ can be explained by other interactions with the column material due to the modified structure or a fractionation during the purification. The structure of the GEDAC4-PEG4-biotin adduct was characterized by ^13^C NMR spectroscopy. In comparison, the ^13^C NMR spectra of GEDAC4 and the biotin-functionalized GEDAC4 are shown in Figure 4. The spectrum of the GEDAC4-PEG4-biotin adduct shows new signals for both amide carbon atoms at 177 ppm (C11, 22). The signals of the PEG chain are observable at 70–68 ppm (C13–20), and the signals of the biotin structure are visible at 62–25 ppm (C23–30). The assignment of the biotin signal was taken from the literature [37].

### 3.4. Binding Capacity

**Figure 5:**
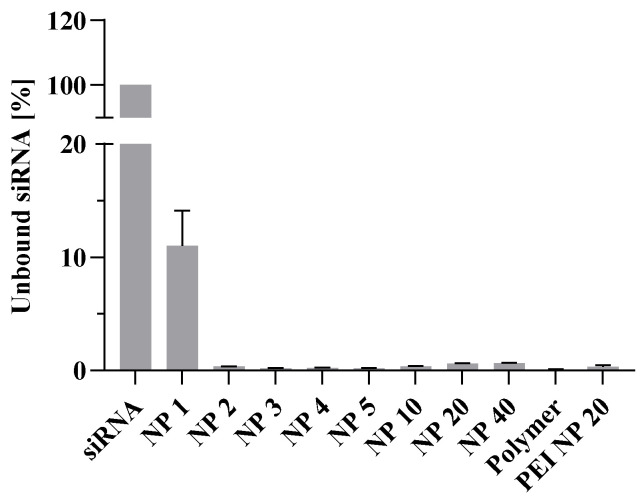
Binding capacity of the dually modified cellulose was investigated using the Quant-iT™ microRNA Assay Kit. N/P ratios between 1 to 40 were evaluated in saline buffer. Free *HDAC3* siRNA served as 100% control. Branched poly(ethylene imine) at N/P ratio 20 worked as the positive control (PEI NP 20). A polymer solution was included to evaluate possible interactions with the assay. Results are shown as mean ± SEM (*m* = 4, *n* = 2; 4 technical replicates per experiment in 2 independent experiments).

To investigate the physicochemical properties, polyplexes of different N/P ratios were formed and the binding affinity of the cellulose was determined by a fluorescent dye-exclusion assay. Free *HDAC3* siRNA was applied as the 100% control demonstrating the full extent of the intercalating dye, and, therefore, the highest fluorescent signal was recorded. Possible unspecific interactions were excluded by introducing a polymer control. Branched PEI at N/P ratio 20 functioned as the positive control, because PEI has a high binding affinity [38]. This assay revealed the high binding affinity of the polymer starting with N/P ratio 2 (Figure 5). At N/P ratio 1, around 10% of siRNA were unbound due to the low polymer content and few electrostatic interactions. Higher N/P ratios obtained the same high binding affinity as the positive control. 

Results were confirmed by agarose gel electrophoresis (Figure S5). For horizontal agarose gel electrophoresis, polyplexes were loaded onto a gel with naked siRNA as control to visualize unbound siRNA in the different N/P ratios. A band of free siRNA occurred for N/P ratio 1, comparable with the lane of free siRNA. However, free siRNA could not be observed for higher N/P ratios. Because of the cationic charge of the polyplexes and, therefore, the masking of the anionic charge of siRNA, the material remains in the gel pockets. The increase of the binding efficacy is well known to correlate with the increasing polymer content at higher N/P ratios [39,40].

To examine the protection ability of the polymer, the polyplexes were treated with RNase A, which was inactivated afterwards with DEPC. To release the undegraded siRNA, the samples were mixed with heparin, which serves as a competitor due to its negative charge similar to siRNA. RNase exposure or RNA release by heparin served as controls. The enzyme-treated siRNA was completely degraded, lacking a characteristic band in the gel, in comparison to naked siRNA and siRNA treated without the enzyme. Both RNA controls without enzyme remained intact, excluding a possible degradation through the treatment. Due to its low binding efficiency, N/P ratio 1 was not investigated by heparin-release assay. The examined N/P ratios from 2 to 40 showed unaffected siRNA after the treatment and heparin release, indicating a protection against enzymatic degradation. Even after treatment with a higher RNase A concentration, the protection against the degradation remained unaltered (Figure S6). The 2′-hydroxyl group of siRNA, which distinguishes it from DNA, is responsible for the higher sensitivity to nucleases and hydrolysis [41]. Often, chemical modifications of the siRNA or PEGylation of the carrier are required to obtain sufficient protection against nucleases [42]. However, the investigated polymer already provides adequate protection, without further modification either of the siRNA or the polymer.

Investigating the binding capacity of the polymer in four different media to find possible distinctions for the use as a transfection medium demonstrated no differences between the FCS-free media (Figure S7). The amount of free siRNA is almost equal for all tested N/P ratios in water, saline buffer, and Opti-MEM^TM^ and is comparable to the positive control. Only in RPMI 1640 + 10% FCS did the amount of free siRNA rise up to 27% for N/P 5, and the values for the higher N/P ratios increased slightly as well due to the formation of a protein corona and interactions with serum proteins [43]. Briefly, the polymer is unable to complex all the siRNA under serum conditions. Consequently, FCS-containing media are not qualified to be used as polyplex-formation buffers for transfection experiments. Once polyplexes have been formed, they can be added to cells in FCS-containing medium.

### 3.5. Particle Size and Zeta Potential

**Figure 6:**
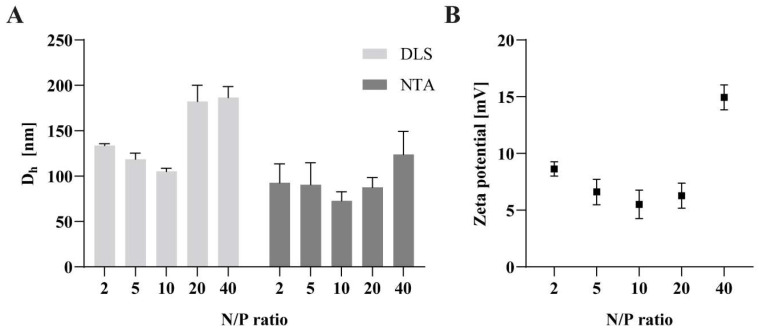
Physicochemical properties of polyplexes. (**A**) Size of polyplexes with N/P ratios between 2 and 40. Size measurements were performed with either dynamic light scattering (DLS) or nanoparticle tracking analysis (NTA). (**B**) Zeta potential of polyplexes with N/P ratios between 2 and 40. Zeta potential was determined via laser doppler anemometry. Data are shown as mean ± SEM (*n* = 3).

The DLS measurements reported a hydrodynamic diameter (D_h_) of the polyplexes between 100 and 130 nm for N/P ratio 2 to 10. Higher N/P ratios demonstrated sizes around 185 nm (Figure 6). The results obtained via the nanoparticle tracking analysis show particle sizes around 90 nm for N/P ratio 2 to 20. Only N/P ratio 40 revealed a size of 125 nm. The size variations between those two measurement techniques were previously reported in the literature and can be attributed to the higher resolution of the NTA measurements compared to the DLS measurements [44]. The increase of the size at N/P ratio 20 and 40 can be explained by an excess of the polymer, which is no longer required to complex the siRNA. Despite the increase of the particle size, the polyplexes were still able to complex the siRNA, as shown by the results of the complexation assay. All investigated N/P ratios formed nanoparticles in size ranges below 200 nm, suggesting their suitability for cellular uptake [45,46].

For the zeta potential measurements, aqueous solutions of N/P ratio 2 to 40 were prepared. The polyplexes exhibited a zeta potential below 10 mV for N/P ratio 2 to 20 and around 15 mV for N/P ratio 40 (Figure 6). This increase of the zeta potential for the highest N/P ratio results from the increasing polymer ratio and, thus, an increase of the positive charge. A positive zeta potential should lead to a sufficient cellular uptake because of electrostatic interactions with negatively charged groups (e.g., sulphate groups) on cell membranes [47].

### 3.6. Endolysosomal Stability

**Figure 7:**
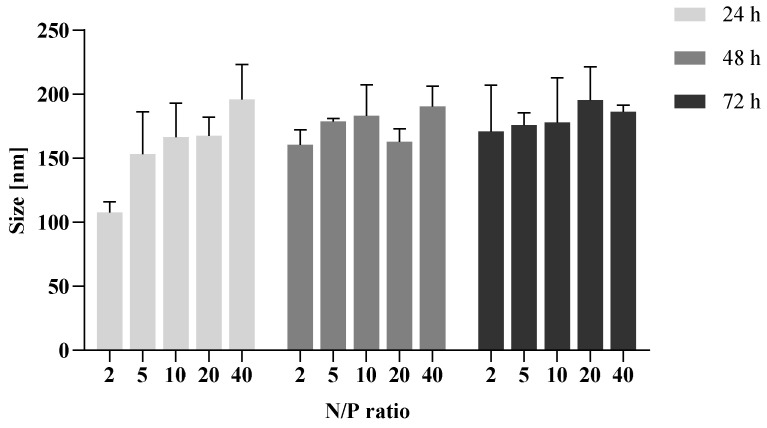
Particle size changes were measured to study the endolysosomal stability of polyplexes, which were incubated under acidic conditions (pH = 4; 37 °C). Size was determined via NTA. Data are shown as mean ± SEM (*n* = 2; 5 technical replicates per experiment).

To evaluate the endolysosomal stability of nanoparticles, polyplexes were prepared in aqueous solutions and the pH value was adapted to 4. The polyplexes were stored for 24, 48, and 72 h at 37 °C to mimic intracorporal conditions. Investigating particle sizes afterwards revealed only slight increases in the particle sizes by around 50 nm, compared to the size measurements in neutral saline buffer. This phenomenon can be explained due to the protonation of the polymer under acidic conditions and, hence, swelling of the particles [48]. No reduction of the particle sizes could be observed (Figure 7), indicating no degradation of the particles over this period and a stable encapsulation of the siRNA.

The physicochemical characterization of the polyplexes proved that they are a suitable complexation system for siRNA because of the high binding affinity independent of the used buffer, the ability of protection against enzymatic degradation, a particle size below 200 nm, a positive zeta potential, and resistance against low pH values for over up to 72 h.

### 3.7. Cell Viability Assay

**Figure 8:**
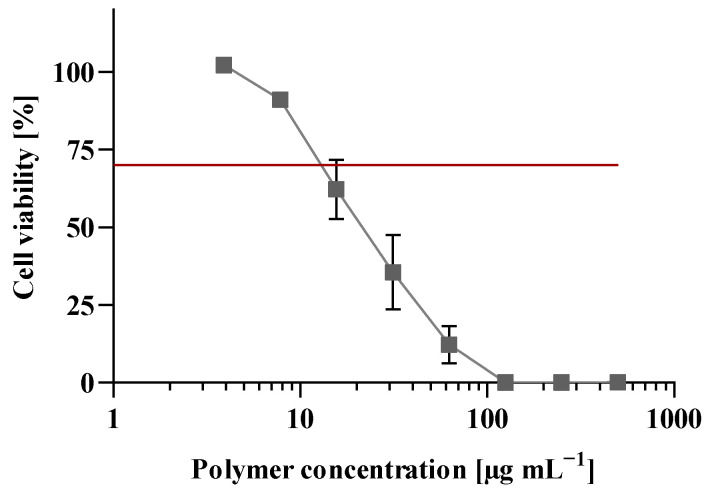
Effect of polymer concentrations between 3.9 to 500 µg mL^−1^ on cell viability. L929 mouse fibroblasts were treated for 24 h, and cell viability was measured by CellTiter-Glo^®^-Assay. Untreated cells were set as 100%. The red line represents the threshold of 70%, according to DIN EN ISO 10993-5. Experiments were performed in quadruplicates. Data are shown as mean ± SEM (*n* = 3).

For biocompatibility testing and to exclude a toxic effect of the polymer, L929 mouse fibroblasts were selected according to DIN EN ISO 10993-5 [36]. Untreated cells functioned as 100% cell viability control and thiomersal 0.02% as the positive control, exhibiting high cytotoxicity [49]. After polymer treatment, L929 cells displayed high cell viability (>70%) for concentrations below 10 µg mL^−1^ (Figure 8). In the common kidney cell line HEK293, concentrations below 4 µg mL^−1^ resulted in a cell viability of over 70% (Figure S9). According to DIN EN ISO 10993-5, a cell viability of 70% and more is defined as non-toxic [36]. For polyplex formation, concentrations below 10 µg mL^−1^ are used, indicating a high biocompatibility for the used polymer concentrations.

Compared to other cationic-modified polysaccharides [50,51], the obtained polymer shows stronger effects on the cell viability and a lower IC_50_ value. In order to address concerns regarding biocompatibility, the corresponding N/P ratios were, therefore, also tested for their effect on the endothelial barrier.

### 3.8. Effect of Polyplexes on Endothelial Barrier Integrity

**Figure 9:**
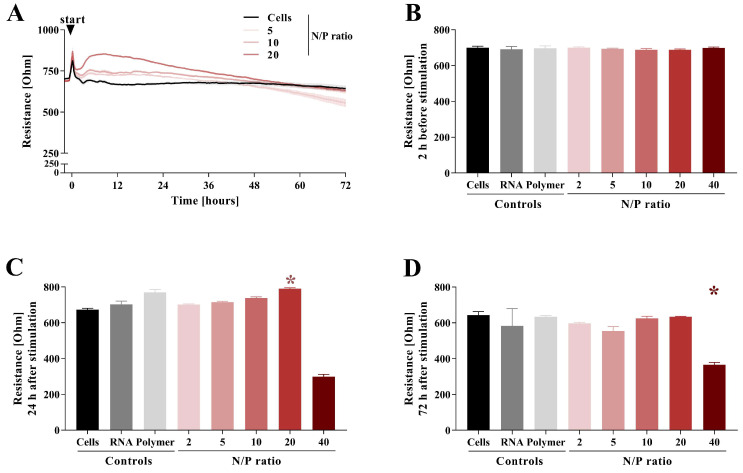
Barrier integrity of human microvascular endothelial cells (HMEC-1) after treatment with polyplexes of N/P ratio 2, 5, 10, 20, and 40. (**A**) Course of resistance at 4 kHz from the time of stimulation until 72 h for cells treated with polyplexes of N/P ratio 5, 10, and 20. (**B**) Resistance at 4 kHz 2 h before stimulation with polyplexes of displayed N/P ratios, free *HDAC3* siRNA (RNA), or free polymer (Polymer). (**C**) Resistance at 4 kHz 24 h after stimulation with polyplexes of displayed N/P ratios, free *HDAC3* siRNA, or free polymer. (**D**) Resistance at 4 kHz 72 h after stimulation with polyplexes of displayed N/P ratios, free *HDAC3* siRNA, or free polymer. Results are presented as mean ± SEM (*n* = 3). * *p* < 0.05 compared to control (Cells). Kruskal–Wallis and Dunn’s multiple comparison test.

In order to test whether the polyplexes influence or disrupt the endothelial barrier integrity, HMEC-1 were monitored using ECIS^TM^. The resistance was measured at 4 kHz (Figure 9) to determine the endothelial barrier integrity, whereas capacitance was measured at 64 kHz (Figure S10) to determine cell detachment as a sign of cytotoxicity and cell death.

After HMEC-1 reached a steady-state resistance (Figure 9B), the cells were stimulated with free *HDAC3* siRNA, polymer, or polyplexes of the displayed N/P ratios (Figure 9), and the measurement was continued for 72 h.

Within the first 24 h after stimulation, N/P ratio 20 induced an initial increase in resistance (Figure 9C) and a decrease in capacitance (Figure S10C). This effect of initial resistance increase has already been described for ECIS studies of bacterial infection and the cytotoxicity testing of nanoparticles [52,53]. However, the underlying mechanisms are not fully understood. In the experimental setup we chose, the slightest morphological changes of the cells during uptake and endosomal processing could induce a change in resistance and capacitance, which could be due to the higher D_h_ and zeta potential of polyplexes with N/P ratio 20 and 40. In addition, different charges of polyplexes dependent on the N/P ratio could also influence the resistance and capacitance measures. After the initial increase in resistance and a reciprocal decrease in the capacitance measure induced by N/P ratio 20 after 24 h (Figure 9C and Figure S10C), both returned to the average level of the untreated control after 72 h (Figure 9D and Figure S10D). The resistance dropped in cells treated with N/P ratio 40 to a significantly lower average resistance compared to the untreated control after 72 h, whereas the capacitance increased reciprocally (Figure 9D and Figure S10D). Stimulation with free *HDAC3* siRNA, polymer, and polyplexes of N/P ratio 2, 5, and 10 neither induced a change in resistance nor in capacitance after 72 h compared to the untreated control (Figure 9D and Figure S10D). All in all, N/P ratio 40 induced not only the breakdown of the endothelial barrier but revealed to be cytotoxic to endothelial cells, whereas the remaining N/P ratios neither broke down the endothelial barrier nor seemed to be cytotoxic to the endothelial cells, allowing their application for further purposes, with exception of N/P ratio 40. A similar concentration-dependent effect has been found for chitosan, an amino polysaccharide [54]. Nanomaterials have been shown previously to interrupt the endothelial barrier, a mechanism described as nanomaterial-induced endothelial cell leakiness (NanoEL). While this effect can be preferable for anti-cancer medications, in other cases, it can raise health risks for the patient, highlighting the importance of this experiment [55,56].

### 3.9. Polyplex Transfection

**Figure 10:**
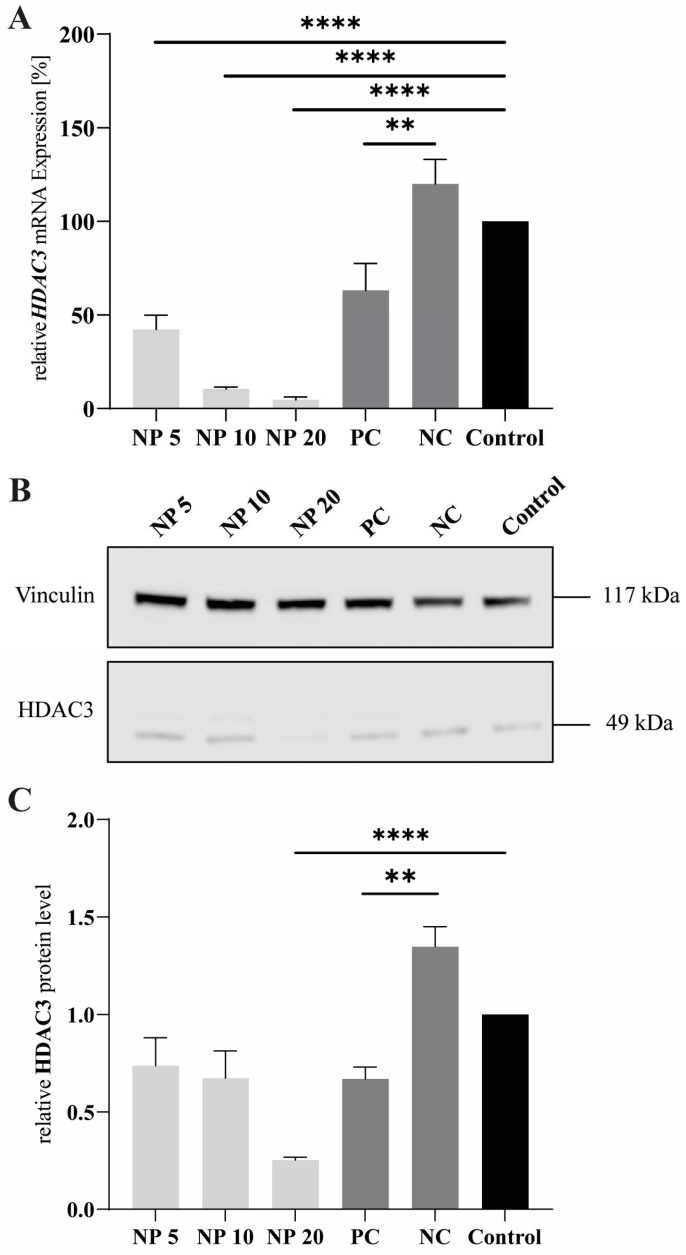
HDAC3 gene and protein expression after 72 h of transfection with polyplexes. *HDAC3*-siRNA-bearing Lipofectamine 2000^TM^ served as positive control (PC) and *GFP*-siRNA (NC) was used as negative control. Untreated cells were used as control. (**A**) *HDAC3* gene knockdown efficiency in HEK293 cells using RT-qPCR. *Rps2* was used as housekeeping gene and *HDAC3* levels were normalized to the *Rps2* level. The results were evaluated using the 2^−ΔΔct^ method. Samples were measured as triplicates. (**B**) HDAC3 protein levels in HEK293T cells after transfection. Vinculin was used as internal control protein. (**C**) Quantification of protein levels. The results were quantified using the Fusion software. Experiments were performed thrice. Results are presented as mean ± SEM (*n* = 3). The *p*-value was set at *p* < 0.05 (** *p* ≤ 0.01, **** *p* ≤ 0.0001).

For the gene expression analysis and the determination of the transfection efficiency, HEK293 cells were transfected with polyplexes bearing *HDAC3* siRNA for 72 h and Lipofectamine 2000^TM^ with *HDAC3* siRNA as transfection reagent control or with *GFP*-siRNA as the negative control. The transfection with the polyplexes resulted in a significant knockdown of *HDAC3* mRNA levels for all analyzed N/P ratios. N/P ratio 5 already provided a knockdown down to 40%, whereas N/P ratio 20 could reach a remaining *HDAC3* expression of around 5% (Figure 10). This N/P ratio-dependent transfection efficiency was reported as well for chitosan-based delivery systems [57]. The positive control lipofectamine only achieved a knockdown to 60% gene expression, indicating potential issues of the lipid complexes to efficiently encapsulate and deliver siRNA or to escape the endolysosomal pathway. These results confirm the high potential of the dually modified cellulose as a non-viral vector for siRNA delivery.

To determine the HDAC3 protein levels after nanoparticle treatment, a Western blot analysis of cell lysates after 72 h was performed. HEK293T cells were treated as described above. Figure 10A shows a clear reduction of the protein signal from N/P ratio 20, stronger than for the positive control (PC), and, through quantification, this reduction could be confirmed as significant compared to the control. For the positive control, N/P ratio 5 and 10, only a slight reduction of the band is visible in comparison to the control, but, nevertheless, the reduction obtained through N/P ratio 10 is significant (Figure 10B). N/P ratio 10 presented almost the same effect as the positive control, whereas N/P ratio 20 performed better, with an almost twofold reduction compared to the lipofectamine effect. The results of the Western blot confirm the results of the RT-qPCR, thus reiterating the great potential of this delivery system. The biotin group presumably has a positive effect on the transfection efficiency by allowing interaction of the polyplexes with the biotin receptor. The enhanced uptake of particles by this mechanism has already been described in the literature [58,59].

### 3.10. Immunofluorescence

**Figure 11:**
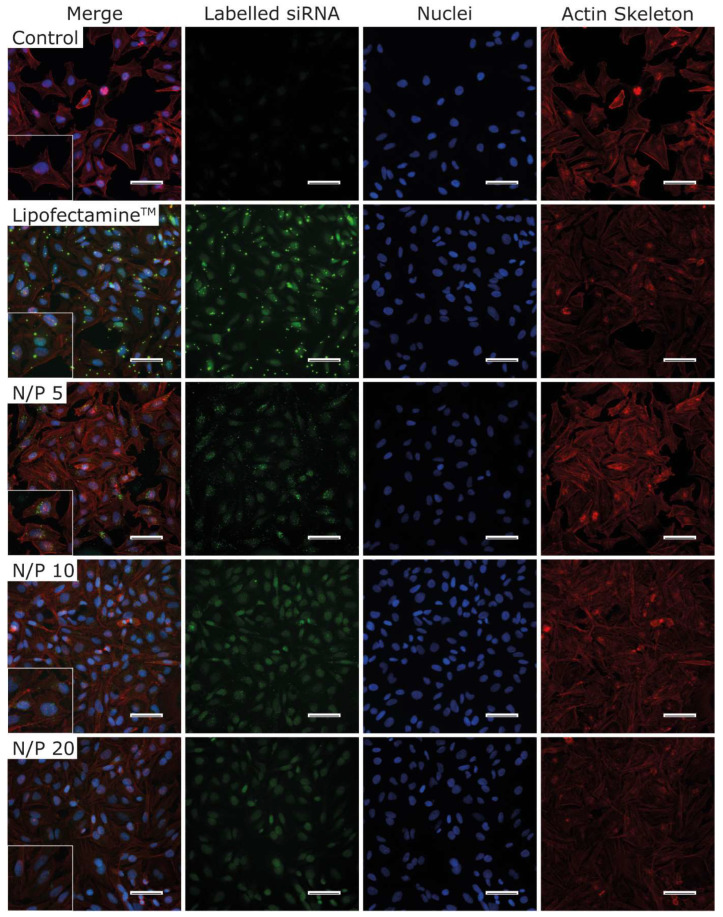
Immunofluorescence of HeLa cells after 90 min of incubation with polyplexes of N/P ratio 5 to 20 containing FAM-labelled siRNA. The Lipofectamine control consisted of FAM-labelled siRNA and Lipofectamine 2000^TM^. The control shows untreated cells omitting labelled polyplexes. Nuclei are stained in blue (DAPI), and the actin skeleton is stained in red (rhodamine). Scale bar: 100 µm. Enlarged section shows an additional 2× magnification.

To visualize the uptake of the polyplexes, FAM-labelled siRNA was used to generate polyplexes with N/P ratio 5, 10, and 20. HeLa cells were incubated with these polyplexes for 90 min and afterwards stained with DAPI and rhodamine-phalloidin. Lipofectamine 2000^TM^ was used as the positive control, and cells treated without nanoparticles served as negative control. Comparing the polyplexes to the Lipofectamine lipoplexes, the size differences are evident. In addition, less fluorescence per cell is visible for the lipoplexes in comparison to the polyplexes, of which several polyplexes are visible inside of the cytoplasm. For N/P ratio 5, the polyplexes appear as small, punctuated pattern, indicating that the polyplexes remain in the endosome and are not released after 90 min for this N/P ratio compared to higher N/P ratios. The same pattern is visible for N/P ratio 10 but, in addition, there is also a diffuse green distribution recognizable (Figure 11). A mixture of a diffuse distribution and endosomal-encapsulated lipoplexes is also visible for the Lipofectamine^TM^ control. This reveals a partial release of siRNA from the endosome to the cytoplasm, comparable to a calcein assay [58]. The intensity of the diffuse distribution increases again for N/P ratio 20, which implies an even higher amount of released polyplexes from the endosome. This effect occurs probably due to the higher polymer amount, which allows stronger interactions with the endosomal membrane and, therefore, a faster release of the polyplexes compared to lower N/P ratios. The presumed mechanism behind the endosomal release of the polyplexes is justified in the proton sponge effect, which causes a protonation of the polymer in the endosome because of its buffering capacity. Consequently, the osmotic pressure in the endosome rises until the endosome ruptures and releases the polymer and its cargo [60]. The siRNA seems to translocate into the nucleus, potentially due to the effect that siRNA accumulates to their target RNA sites and HDAC3 is expressed in the nucleus [61,62]. Furthermore, Gagnon et al. discovered RNAi factors like Dicer in the nucleus, which presumably leads to siRNA accumulation in the nucleus [63].

## 4. Conclusions

This study describes the synthesis of a new non-viral vector based on a biotinylated guanylated cellulose derivative. Thus, 2-aminoethylamino celluloses with a DS_EDA_ of 0.54 and 0.81 were guanylated by the reagent PCA in an aqueous NaOH solution under mild reaction conditions. The amino moieties were converted to guanidinium groups. The efficiency of the amino substituent conversion ranged from 46% to 85%. The characterization of the structure was performed by ^13^C NMR spectroscopy, which revealed the guanylation of the secondary amino moiety at a reaction temperature of 80 °C. Subsequently, the modification of the remaining primary amino groups was possible with the active ester biotin (PEG4)-linker NHS ester, which was added to enable the binding of targeting ligands. The cellulose backbone provides the advantage of a biodegradable, sustainable, and easily producible resource compared to synthetic alternatives like polymethacrylamides and polyethyleneimines [13,64]. 

The dually modified cellulose was able to form polyplexes together with *HDAC3*-siRNA. The physicochemical characterization revealed nanosized, positively charged nanoparticles with a very high RNA-binding efficiency, starting with N/P ratio 2. This binding capacity resulted in an effective protection against RNase treatment and acidic, endolysosomal conditions. In vitro investigations revealed its high potential as a transfection vector through its moderate toxicity in L929 cells paired with no influence on the endothelial barrier integrity, measured by ECIS^TM^. This compares favorably to other nanomaterials, which exhibit the NanoEL, indicating the use of these polyplexes for diseases like the hemolytic uremic syndrome (HUS), which is characterized through a damaged endothelium in humans. The uptake and endosomal release could be demonstrated by immunofluorescence in HeLa cells after 90 min. The superior efficiency as a non-viral vector compared to commercially available transfection reagents like Lipofectamine 2000^TM^ was confirmed at gene expression and protein levels, where the expression of HDAC3 was significantly downregulated.

In conclusion, this vector carries the immense potential for comprehensive applications in the field of gene delivery. Here, the biotin moiety opens new areas of use due to the possibility of coupling any desired targeting ligand, e.g., peptides and antibodies, via the biotin streptavidin system. Hence, organs can be specifically targeted. Also, biotin itself can act as a targeting ligand to promote nanoparticle delivery and to enhance cellular uptake [58,65]. Taken together, the results reveal the great potential of these cellulose-based polyplexes to be used for the targeted therapy of a variety of diseases with an adaptable treatment approach. Further studies will focus on coupling with targeting moieties and the investigation of the treatment of inflammatory diseases with these polyplexes.

## Figures and Tables

**Table 1:** Reaction conditions and results of the conversion of 6-deoxy-6-(2-aminoethyl) amino cellulose with 5 mol of 1*H*-pyrazol-carboxamidine hydrochloride per mol modified RU in aqueous NaOH for 24 h.

Conditions		Sample	Results	
DS_EDA_	T [°C]		Degree of Guanylation DG ^(a)^	Conversion of EDA [%]
0.54	60	GEDAC1	0.25	46.3
0.54	80	GEDAC2	0.33	61.1
0.81	80	GEDAC3	0.62	76.5
0.81	80	GEDAC4	0.69	85.2

^(a)^ DG determined by conductometric titration.

## Data Availability

Data are contained within the article or Supplementary Materials. Raw data and materials are available from the authors upon reasonable request.

## Supplementary Materials

The following supporting information can be downloaded at: https://www.mdpi.com/article/10.3390/pharmaceutics15122659/s1, Figure S1: Conductometric titration graph of 6-deoxy-6-(2-guanidiniumethyl) amino cellulose GEDAC2; Figure S2: ^13^C NMR spectra of (A) 6-deoxy-6-(2-guanidiniumethyl) amino cellulose GEDAC1, GEDAC2, and GEDAC3; Figure S3: HSQC DEPT NMR spectrum of 6-deoxy-6-(2-guanidiniumethyl) amino cellulose GEDAC2; Figure S4: HMBC NMR spectrum of 6-deoxy-6-(2-guanidiniumethyl) amino cellulose GEDAC2; Figure S5: (A) Agarose gel electrophoresis of polyplexes, (B) Agarose gel electrophoresis after RNase A treatment; Figure S6: RNase A treatment with different concentrations; Figure S7: Binding efficiency of the polymer in different media; Figure S8: Determination of the biotin amount in different N/P ratios; Figure S9: Cell viability of HEK293 cells; Figure S10: Cell viability of HMEC-1 after polyplex treatment.
