# Supplementary material for: Dually Modified Cellulose as a Non-Viral Vector for the Delivery and Uptake of HDAC3 siRNA
Source: Pharmaceutics. 2023 Nov 23;15(12):2659. doi: 10.3390/pharmaceutics15122659 (PMC10747125; doi:10.3390/pharmaceutics15122659)
Supplement: Supplementary file 1 [file pharmaceutics-15-02659-s001.zip › pharmaceutics-2709149-supplementary.pdf]

Supporting Information

1. Experimental Section

1.1. Agarose gel electrophoresis

For gel electrophoresis, 50 µL of different N/P ratios were prepared and mixed with 5 µL of loading buffer (40 mM Tris, 50 % glycerol (85 %), 1 mM ethylenediaminetetraacetic acid (EDTA), all from Carl Roth) pH 7.4. Electrophoretic separation was carried out using a 1 % agarose gel (peqGold Universal Agarose, Peqlab Biotechnology, Erlangen, Germany) with 0.125 µg mL^{-1} ethidium bromide (SERVA Electrophoresis GmbH, Heidelberg, Germany). Free siRNA served as positive control. The separation took place in an electrophoresis chamber (Biometra, Goettingen, Germany) for 1 h with TAE running buffer (40 mM Tris, 1 mM EDTA, 0.1 % acetic acid, all Carl Roth). Gel photos were received using UV transillumination (Inas GmbH, Goettingen, Germany) at 312 nm and evaluated with the software BioVision (VILBER, Collegien, France).

Enzymatic stability of the polyplexes was investigated after incubation of polyplexes with 0.4 µL RNase A (1.5 units μg^{-1} siRNA, ThermoFisher™) for 45 min at 37 °C. RNase A was inactivated by adding 0.5 µL of diethylpyrocarbonate (DEPC) (Carl Roth). The intact siRNA was released by heparin (20 I.U., Carl Roth) treatment of the samples for 20 min at 37 °C. For visualization of the remaining intact siRNA, an agarose gel electrophoresis was performed as previously described. Untreated siRNA, siRNA equally treated but without addition of RNase A and RNase A treated siRNA were used as siRNA control, negative and positive control.

1.2. Protection against enzymatic degradation using different concentrations

This experiment was performed as the publication describes. The concentration of the RNase A was adapted to 3 U μg^{-1} siRNA and 4.5 U μg^{-1} siRNA.

1.3. Quantification of the biotin content in polyplexes

For the biotin quantification polyplexes of N/P ratio 5 , 10 and 20 were formed in water as described in the manuscript. The Pierce™ Fluorescence Biotin Quantitation Kit (ThermoFisher™) was used for quantification according to the manufacturer instructions. A Tecan® Spark 20M (495 nm excitation, 520 nm emission, Tecan Group AG) was used to capture fluorescence. For the standard curve a biocyctin standard (0-100 pmol 10 μL^{-1}) was used and with this the biotin content of the N/P ratios was calculated. Samples were measured in triplicates, the assay was repeated twice (n=3). Data are displayed as mean.

1.4. Cell Viability Assay on HEK293 cells

For investigation of the polymer's cytotoxicity in HEK293 cells, 10,000 cells per well were seeded into a 96 well-plate (Greiner Bio-One) and incubated for 24 h. The polymer dilution row was prepared and pipetted as described in the publication. After

another 24 h of incubation, the polymer solutions were aspirated and replaced through a 500 µg mL^{-1} solution of 3-(4,5-dimethylthiazol-2-yl)-2,5-diphenyltetrazolium bromide (MTT) (Alfa Aesar, Haverhill, USA). These cells were incubated for 4 h followed by removal of the MTT solution and dissolving the dye in isopropanol (Carl Roth, Karlsruhe, Germany). The plate was shaken for 10 min to solve the dye, following absorbance measurement at 570 nm utilising a FLUOstar OPTIMA Platereader (BMG Labtech, Ortenberg, Germany). Untreated cells served as negative control and were set as 100 %, a thiomersal 0.02 % solution served as positive control (Caelo, Hilden, Germany). To calculate the cell viability, the blank was subtracted from the samples and the percentage of the resulting values compared to the negative control was calculated. Pursuant to DIN EN ISO 10993-5 (10993–5, 2009), cell viabilities above 70 % were identified as non-toxic. Samples were prepared with 8 technical replicates and the assay was performed twice. Data are shown as percentages (mean ± SEM).

2. Figures

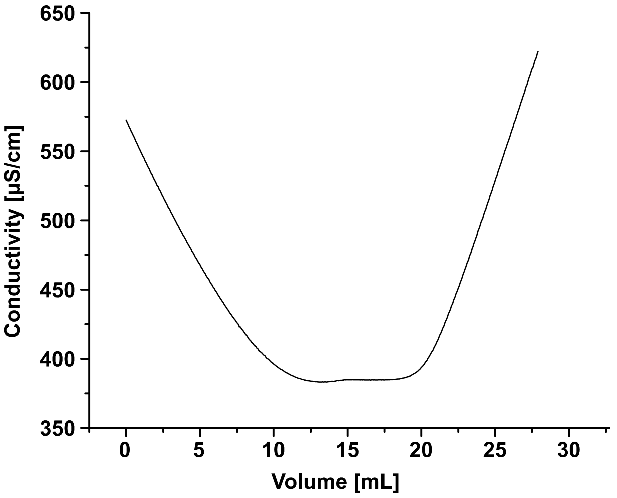

Figure S1. Conductometric titration graph of 6-deoxy-6-(2-guanidiniumethyl) amino cellulose GEDAC2 (DS_{EDA} = 0.54; DG = 0.33).

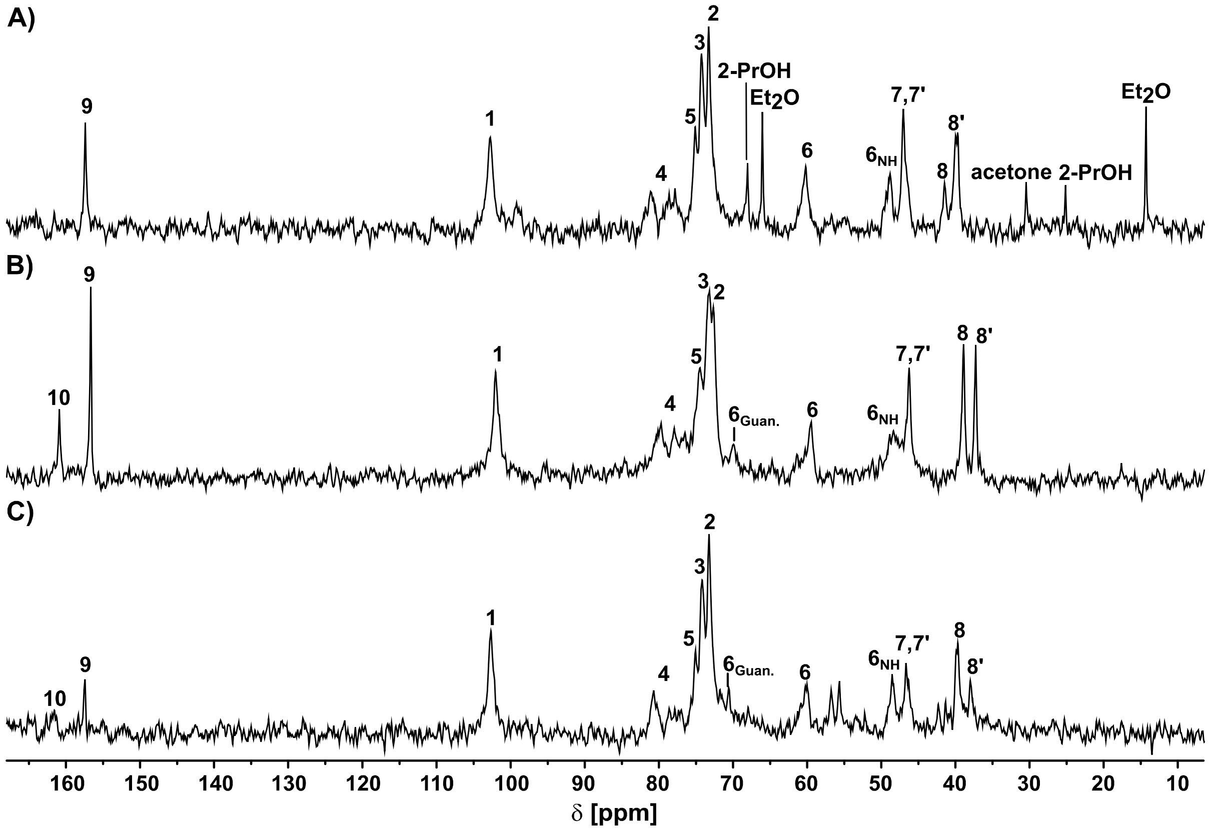

Figure S2. ^{13}C NMR spectra of **A)** 6-deoxy-6-(2-guanidiniumethyl) amino cellulose GEDAC1 (DS_{DAEA} = 0.54; DG = 0.25), **B)** GEDAC2 (DS_{DAEA} = 0.54; DG = 0.33), and **C)** GEDAC3 (DS_{DAEA} = 0.81; DG = 0.62) in D_2O.

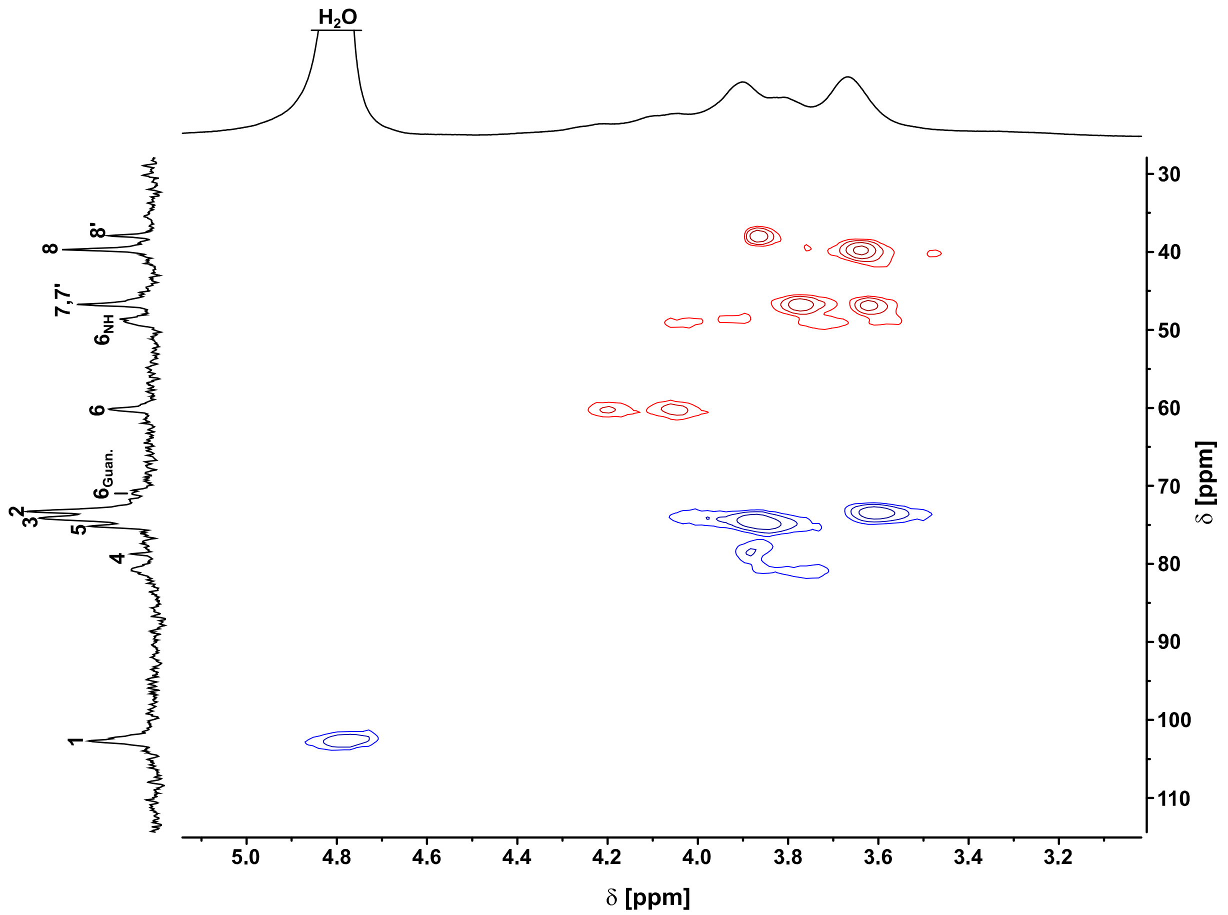

Figure S3. HSQC DEPT NMR spectrum of 6-deoxy-6-(2-guanidiniumethyl) amino cellulose GEDAC2 (DS_{EDA} = 0.54; DG = 0.33) in D_2O.

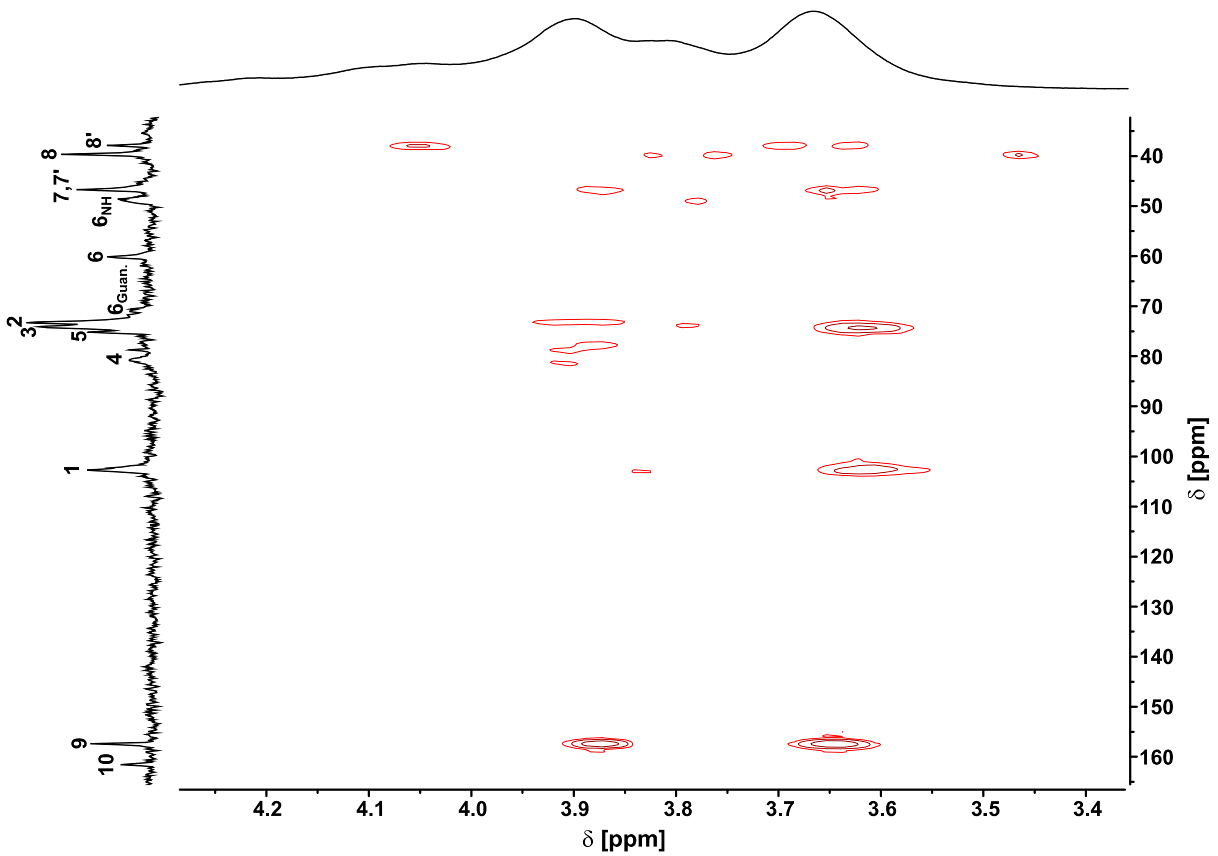

Figure S4. HMBC NMR spectrum of 6-deoxy-6-(2-guanidiniumethyl) amino cellulose GEDAC2 (DS_{EDA} = 0.54; DG = 0.33) in D_2O.

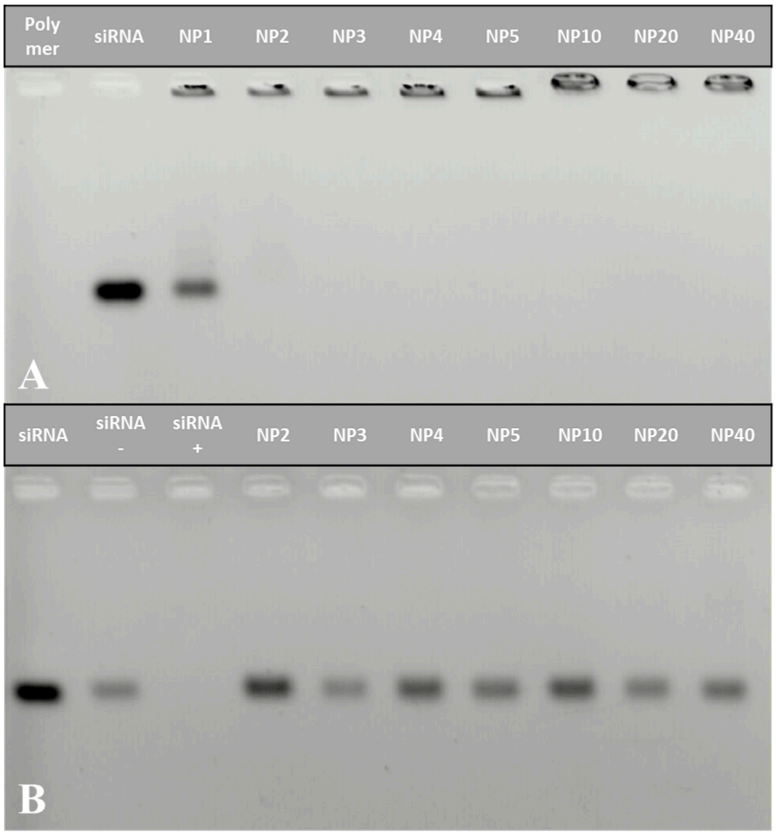

Figure S5. A) Agarose gel electrophoresis of polyplexes with N/P ratios from 1 to 40. A polymer solution and naked siRNA served as positive control. **B)** Agarose gel electrophoresis of polyplexes after treatment with RNase A and heparin release of the siRNA. Untreated siRNA was utilized as control (siRNA), RNase A treated siRNA as positive control (siRNA+) and heparin treated siRNA (without RNase A, siRNA-) as negative control.

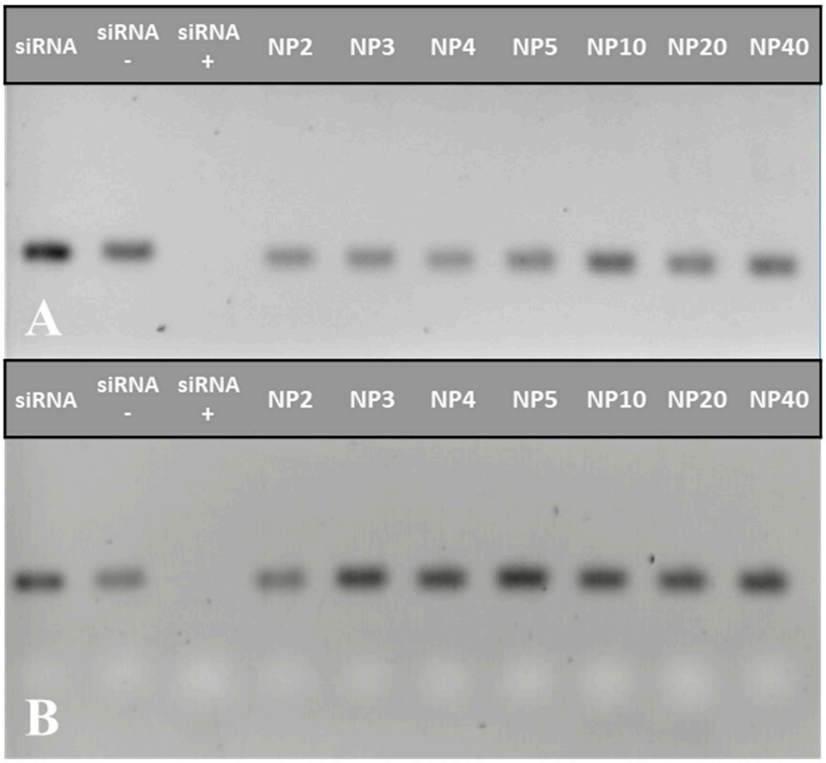

Figure S6. Agarose gel electrophoresis of polyplexes after RNase A treatment with different concentrations. **A)** 3.0 U μg^{-1} siRNA, **B)** 4.5 U μg^{-1} siRNA. Untreated siRNA was utilized as control (siRNA), RNase A treated siRNA as positive control (siRNA+) and heparin-treated siRNA (without RNase A, siRNA-) as negative control.

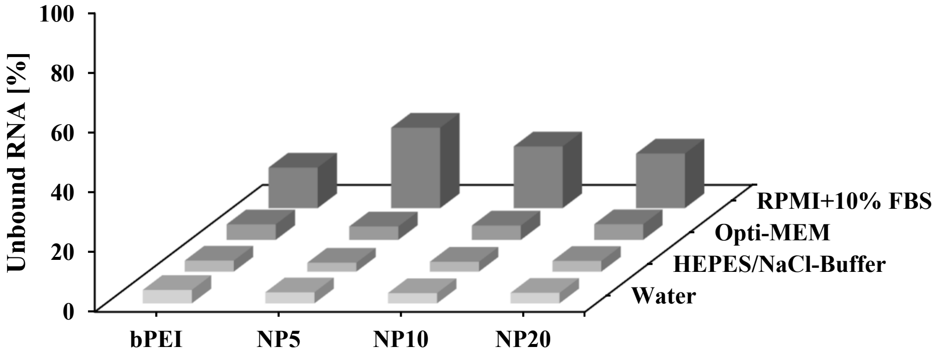

Figure S7. Binding efficiency of the polymer was investigated using the Quant-iT™ microRNA Assay-Kit. N/P ratios between 1 to 40 were assessed in water, saline buffer (HEPES/NaCl-Buffer), Opti-MEM™ or RPMI+10 % FBS. Free siRNA was set as 100 % control (not shown). Branched poly(ethylene imine) at N/P ratio 20 served as positive control (bPEI). Experiment was performed with quadruplicates; results are shown as mean (n = 2).

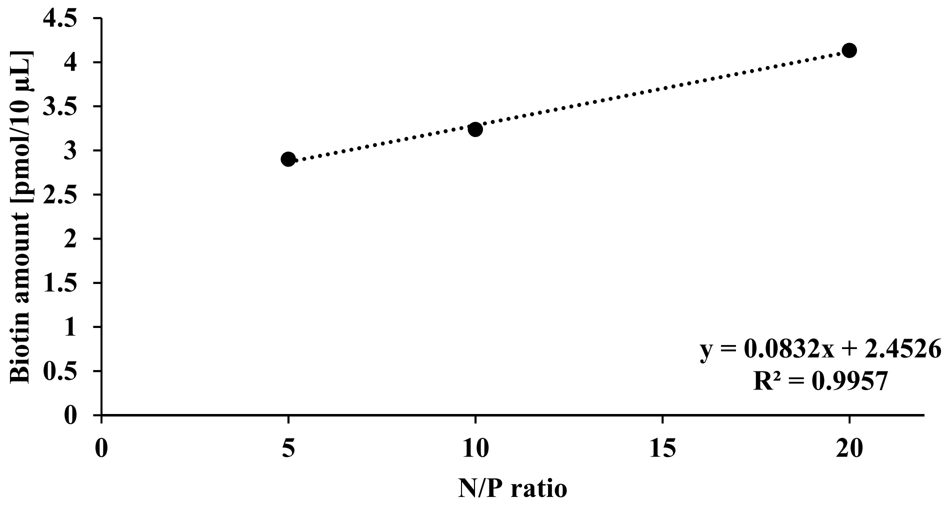

Figure S8. Determination of the biotin amount in different N/P ratios using the Pierce™ Fluorescence Biotin Quantitation Kit. Biotin amount was calculated using a biocytin standard curve. Samples were measured in triplicates and assay was repeated twice (n=3). Data are shown as mean.

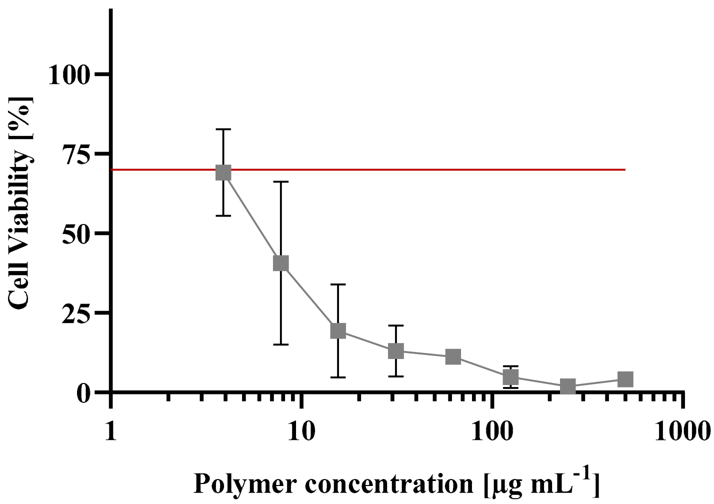

Figure S9. Cell viability of HEK293 cells after polymer treatment. Polymer concentrations between 3.9 to 500 µg mL^{-1} were tested for 24 h. Cell viability was determined with an MTT assay. A thiomersal solution (0.02 %) was used as positive control (not shown) and untreated cells as negative control. The red line represents the threshold of 70 % according to DIN EN ISO 10993-5. Experiments were performed with 8 technical replicates. Data are shown as mean ± SEM (n = 2).

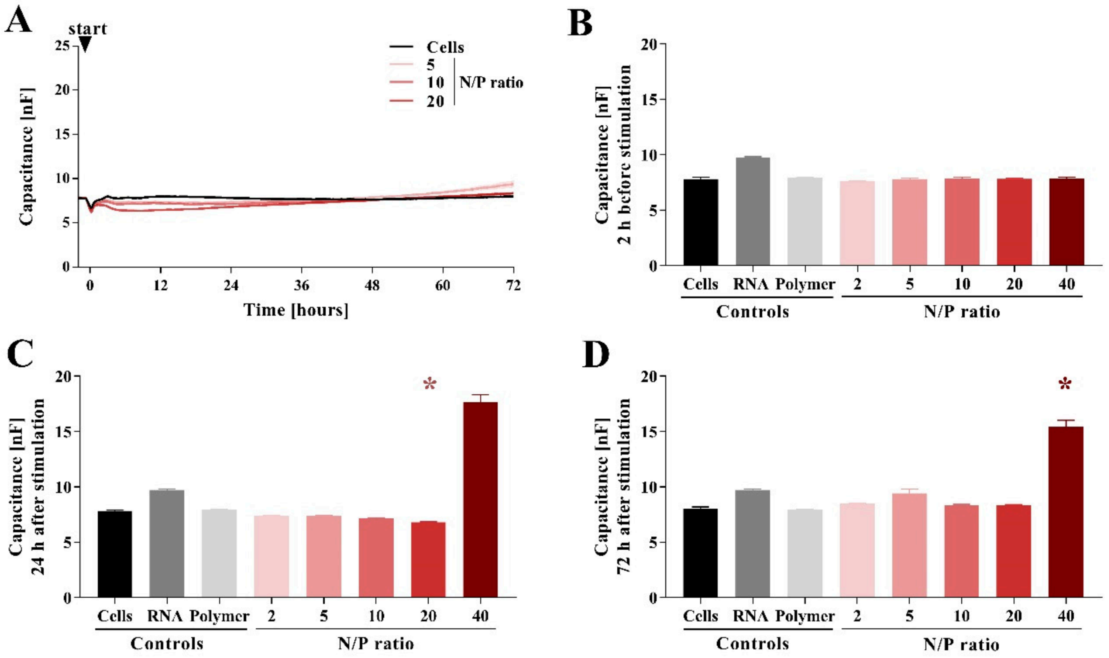

Figure S10. Cell viability of immortalized human microvascular endothelial cells (HMEC-1) after polyplex treatment of N/P ratio 2, 5, 10, 20 and 40. **A)** Course of capacitance at 64 kHz from the time of stimulation until 72 h for cells treated with polyplexes with N/P ratio 5, 10 and 20. **B)** Capacitance at 64 kHz 2 h before stimulation of displayed N/P ratios, free *HDAC3* siRNA or free polymer. **C)** Capacitance at 64 kHz 24 h after stimulation of displayed N/P ratios, free *HDAC3* siRNA or free polymer. **D)** Capacitance at 64 kHz 72 h after stimulation of displayed N/P ratios, free *HDAC3* siRNA or free polymer. Results are presented as mean ± SEM (n = 3). *p < 0.05 compared to control (cells). Kruskal-Wallis and Dunn's multiple comparison test.
